# Supplementary material for: A set of multi-entry identification keys to African frugivorous flies (Diptera, Tephritidae)
Source: Zookeys. 2014 Jul 24;(428):97–108. doi: 10.3897/zookeys.428.7366 (PMC4143993; doi:10.3897/zookeys.428.7366)
Supplement: Supplementary material 6 — Key to Ceratitis [file zookeys-428-097-s006.zip › SF6_ZooKeys_key to Ceratitis/key/SF6_ZooKeys_key to Ceratitis/Media/Html/Ceratitis neostictica .htm]

Ceratitis (Ceratalaspis) neostictica sp


***Ceratitis*** **(*Ceratalaspis*)
*neostictica* De Meyer**

*Ceratitis* (*Ceratalaspis*) *neostictica*
De Meyer, 1998: 283.

Body
length: 4.20 (4.00-4.40) mm; wing length: 4.37 (4.20-4.60) mm.

Male

Head.
Antenna yellow-orange. Third antennal segment three times as long as second
segment. Arista with medium long hairs over entire length. Frons yellow-orange,
with short scattered hairs which are largely the same colour as frons; convex,
in lateral view not distinctly projecting forwards at antennal implant.
Chaetotaxy normal for subgenus.

Thorax.
Postpronotum white, spotted. Ground colour of mesonotum pale with yellow-orange
tinge. Chaetotaxy normal for subgenus. Scapular setae pale. One anepisternal
bristle. Scutellum white basally, otherwise yellow with three black separate markings
apically; basally with two dark separate spots, only slight darker colouration.
Subscutellum completely dark.

Legs
yellow; setation typical for subgenus, mainly pale especially on femora. Fore
femur posterodorsally with bushy pile of yellow-orange hairs; mid tibia and
hind femur at apical third with feathering of same colour. Posterior and
posterodorsal rows on fore femur pale. Ventral bristles on femora pale.

Wing
bands with extensively yellow markings. Banding, cubital band free, marginal
band continuous; medial band free; crossvein r-m at middle of discal cell.
Crossvein dm-cu posteriorly more outwards than anteriorly.

Abdomen.
Yellow. Setation and banding typical for subgenus.

Female

As
male except for the following characters: Medial band not outspoken, at most
weakly present. Legs without feathery or bushy pile. Oviscape shorter than
abdominal terga 3-6 combined.

(Description
after De Meyer, 1998)
